# A Carbon Cycle Model for the Social-Ecological Process in Coastal Wetland: A Case Study on Gouqi Island, East China

**DOI:** 10.1155/2017/5194970

**Published:** 2017-02-14

**Authors:** Yanxia Li, Lihu Xiong, Wenjia Zhu

**Affiliations:** ^1^School of Tourism and Event Management, Shanghai University of International Business and Economics, No. 1900 Wenxiang Road, Songjiang District, Shanghai 201620, China; ^2^Zhejiang Institute of Marine Planning and Design, No. 50 East Fengqi Road, Hangzhou, Zhejiang Province 300201, China; ^3^Faculty of Education, East China Normal University, 3663 North Zhongshan RD, Shanghai 200062, China

## Abstract

Coastal wetlands offer many important ecosystem services both in natural and in social systems. How to simultaneously decrease the destructive effects flowing from human activities and maintaining the sustainability of regional wetland ecosystems are an important issue for coastal wetlands zones. We use carbon credits as the basis for regional sustainable developing policy-making. With the case of Gouqi Island, a typical coastal wetlands zone that locates in the East China Sea, a carbon cycle model was developed to illustrate the complex social-ecological processes. Carbon-related processes in natural ecosystem, primary industry, secondary industry, tertiary industry, and residents on the island were identified in the model. The model showed that 36780 tons of carbon is released to atmosphere with the form of CO_2_, and 51240 tons of carbon is captured by the ecosystem in 2014 and the three major resources of carbon emission are transportation and tourism development and seawater desalination. Based on the carbon-related processes and carbon balance, we proposed suggestions on the sustainable development strategy of Gouqi Island as coastal wetlands zone.

## 1. Introduction

Wetlands are biologically diverse and productive transitional areas between land and water. By occupying zones of transition between terrestrial and marine ecosystems, coastal wetlands, including salt marshes, mangroves, intertidal mudflats, seagrass beds, and shallow subtidal habitats, are the interface of the coastal landscape [1]. Coastal wetlands such as mangroves, salt marshes, intertidal mudflats, and seagrass beds have been suggested to offer many important ecosystem services [2]. Being productive and often spatially diverse habitats, coastal wetlands fulfill important functions such as producing a large variety of food to consumers, providing habitats for flora and fauna including migratory birds, fish, turtles, and cetaceans [3–7], and helping to moderate water quality [8].

Moreover, coastal wetlands zones support a variety of economic activities, including fisheries, aquaculture, tourism, recreation, and transportation. In recent decades, many coastal areas have been heavily modified and intensively developed. Human activities such as waste dumping, land reclamation, aquaculture ponds, and dredging for navigational channels and marinas have resulted in the recent rapid loss of coastal wetland habitats [2, 5]. How to simultaneously decrease the destructive effects flowing from human activities and improve local economic development, thus maintaining the sustainability of regional wetland ecosystems, are an important issue for coastal wetlands zones.

Carbon credit [9] within the area is now considered an effective method during regional sustainability policies making. With carbon credits, we can calculate the carbon emission through human activities and the carbon uptake or removal by natural environmental system such as green plants, algae, and shellfish. Thus the state of “carbon-free” or “carbon neutral” can be considered as the goal for regional sustainable development [10, 11]. To achieve this goal, the carbon emission can be reduced by the introduction of innovative technology, and the carbon removal can be improved by artificial ecological system that can improve or develop new ecosystem service function.

In this article we use carbon credits as the basis for regional sustainable developing policy-making, present the carbon cycle model of Gouqi Island, a typical coastal wetlands zone that locates in the East China Sea, to illustrate the relationship between human activities and natural environment in costal wetland through carbon credits, discuss the regional developing models and paths for Gouqi Island to achieve sustainable development, which can also be generalized to the sustainable development strategy of coastal wetlands zones.

According to the developing strategy of Gouqi Island, tourism has been regarded as one of the major industries in future, together with agriculture (mainly relies on mussel). The island has rich coastline resources; thus tourism activities have witnessed fast development in the past 5 years. Especially after a popular movie named “the continent” was released in 2014 in China, a majority of tourists have come to Gouqi Island to experience leisure in the natural small island. This provides great opportunity for the tourism development in the island. However, as integration of extremely fragile systems, the island is now in heavy demand of sustainable plans that can balance between tourism development, infrastructure building, and tourism activity development through the consideration of economy and environmental conservation through long-term sustainability and natural welfare.

## 2. Study Area and Methods

### 2.1. Study Area

**Figure 1:**
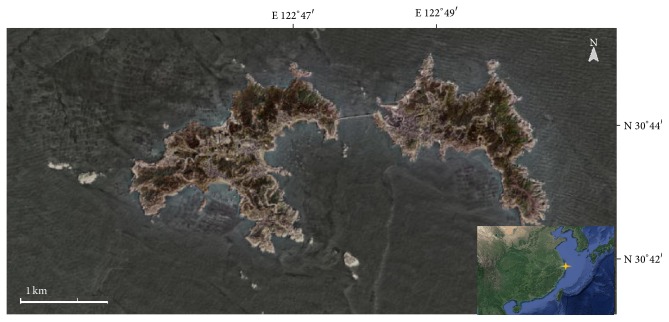
The map of Gouqi Island and its location in East China.

Gouqi Island locates in 30°43′1′′N and 122°46′3′′E where it is in the northeast among Zhoushan Archipelago, it is part of East China Sea coast and Islands wetland ecological system, the map of Gouqi Island is displayed in Figure 1, and the detailed information is listed in Table 1 [12].

The major industries in Gouqi Island are marine fishing industry and marine aquaculture, the marine fishing industries are developed in the area around the island and distant ocean, and the fishing industries are mainly developed along the coast, among which mussel culturing industry contributes around 95% to the industry considering both quantity and economic income. Mussel processing industry is also the traditional and important industry in the island. Besides that, the fresh water in the island relies on the desalination industry; this makes another important industry in the island. The tourism industry in the island has grown fast in the past 5 years and now contributes 45.2% of the GDP for the island. Currently, Gouqi Island receives more than 200,000 tourists per year and some negative impacts have appeared [12]. The energy structure in the island is quite simple, the transportation in the island heavily relies on fossil fuel, and the fossil-fuel plant locating in Gouqi Island provided energy required by desalination and seafood processing industry. The residential electricity is generated by the thermal power plants locating in the Shengsi Island. Besides fishing products, there are no production manufacturers in the island; thus all the food and other materials are imported from outside of the island. Ferry is the only transportation between the island and outside, and transportation in the island relies on transports which use fossil fuels. Because of the narrow road, the main transports on the island are small vehicles, while large trucks or buses cannot be used.

### 2.2. Modelling Methods

Following the model theory proposed by Jørgensen et al. [16] we develop a carbon cycling model based on the socioecological system with STELLA® software; the data used in the STELLA model mainly come from the following: (1) basic information of Gouqi Island from statistics reports; (2) data collecting from the tourists on the island with a questionnaire; (3), coefficients or parameters of processes in the model are cited from literatures; (4) field survey.

## 3. Model Description

### 3.1. Conceptual Model

**Figure 2:**
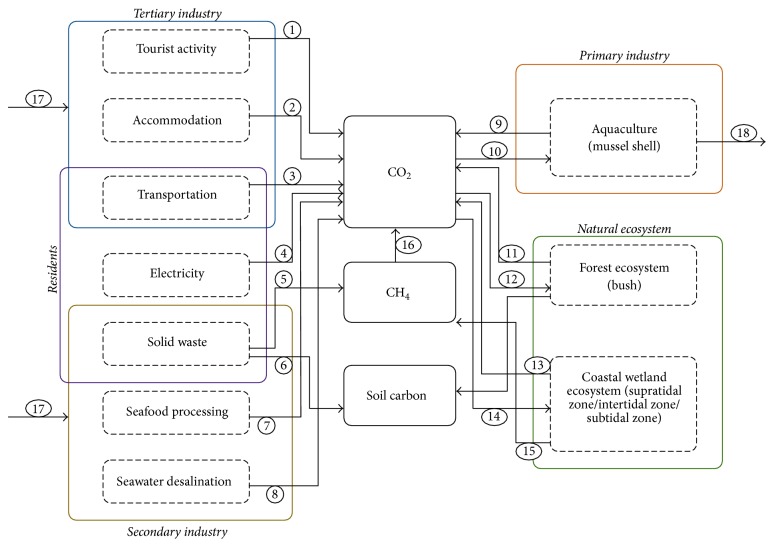
Conceptual model for the social-ecological process carbon cycle model on Gouqi Island. ① Carbon released from tourism activity, ② carbon released from tourism accommodation, ③ carbon released from transportation between island and outside, ④ carbon released of local residents, ⑤ CH_4_ released from solid waste landfill, ⑥ carbon stored by soil carbon pool through solid waste landfill, ⑦ carbon released from seafood processing sector, ⑧ carbon released from desalination of seawater, ⑨ carbon released from marine aquaculture, ⑩ carbon captured by marine aqua-culturing, ⑪ respiration in forest ecosystem, ⑫ photosynthesis in forest ecosystem, ⑬ respiration in wetland ecosystem, ⑭ photosynthesis in wetland ecosystem, ⑮ CH_4_ released from wetland ecosystem, *⑯* oxidation, *⑰* import, and *⑱* harvest.

**Figure 3:**
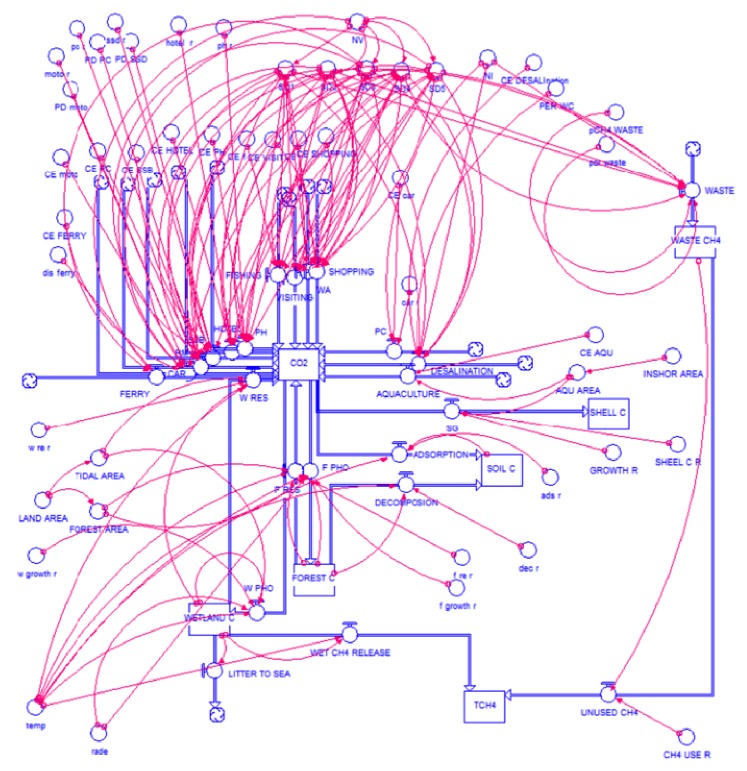
Conceptual STELLA diagram of for the social-ecological process carbon cycle model on Gouqi Island.

We develop a carbon cycle model of Gouqi Island based on the socioecological process that determines the emission of CO_2_ through human activity and economic development and removals of CO_2_ through natural environment process. Jørgensen and Nielsen [17] have developed a carbon cycle model for the Danish island of Samsø to analyze the environmental management policies based on carbon emission and uptake process. We conduct similar procedures to develop a carbon cycle model for Gouqi Island (Figure 2). The main carbon pools, as well as important processes that show the flow of carbon from one pool to another and all the external inputs and outputs of carbon to the island, are reflected in the model. The model includes nature ecosystem (including forest ecosystem and tidal wetland ecosystem), residents (including transportation, electricity, and solid waste), primary industry (aquaculture of mussel), secondary industry (including seafood processing and seawater desalination), and tertiary industry (tourism activity, accommodation, and transportation) [18], and there are some overlaps between residents, secondary industry, and tertiary industry. The main inputs of carbon to the island are the imported food (for residents and tourists), electricity (for residents and tourists use), and fossil fuel. The conceptual model in the STELLA format is shown in Figure 3.

Then we apply data of 2014 to the model, calculate the carbon credits and its components, and discuss possible policies for carbon-neutral target based on scenario analysis under different developing strategy. We use year as the basic unit for the analysis, because both the tourism and natural systems are experiencing periodic development each year. We use tons as the unit when calculating the amount of Carbon.

### 3.2. The Model Components

#### 3.2.1. The State Variables of the Model

The state variables (carbon pools) in the model are expressed the by differential equation following the next format: changes per unit of time equal inputs per unit of time minus outputs per unit of time [19]. The state variables and their symbols in the model are listed in Table 2.

#### 3.2.2. Forcing Functions

The forcing functions or external variables were selected by the development of the conceptual diagram. We list the forcing functions in Table 3 with the symbols used (in STELLA-diagrams a thick arrow with a valve starting or ending with a cloud) in Figure 3.

#### 3.2.3. Processes of the Model

Following the model theory of Jørgensen et al. [16], 24 processes of carbon release and capture ways in social and natural systems of Gouqi Island were described by the development of the conceptual model. Symbols and related units are illustrated in Table 4.

#### 3.2.4. Data Resources of the Model

Published information, questionnaire, and observations by field surveys were used in modelling. Published information is cited from literatures that illustrate similar process or objective, and basic information related to Gouqi Island is cited from the statistic reports. We adopt a Life Cycle Assessment (LCA) questionnaire from Kuo and Chen [13] to collect the tourist data, and the questionnaire includes the following: choice of transportation, choice of accommodation, and activity related to the length of stay (see Appendix); the data are applied directly in the model.  Table 5 shows the summary of the parameter symbols used in the model and the resources of the data.

#### 3.2.5. Process Equations

The processes are described either as zero-order, as first-order, or as Michaelis–Menten equations. Additionally, a logistic growth equation is applied to determine the photosynthetic growth. Basic equations used in the model are illustrated in the following “*Process Equations*”. The processes are expressed in the unit tCyr^−1^.

*Process Equations.* All the equations, parameters, initial values, and forcing functions in the STELLA format are listed in Table 5.

  CO2(t) = CO2(t - dt)+(W_RES+F_RES+ AQUACULTURE+PC+DESALINATION+SHOPPING+ WA+VISITING+FISHING+HOTEL+PH+FERRY+ RM+CAR+SSB - W_PHO - F_PHO - ADSORPTION - SG)^*∗*^dt
  INIT CO2 = 0
  INFLOWS:
  W_RES = w_re_r^*∗*^TIDAL_AREA^*∗*^(20-temp)^*∗*^WETLAND_C
  F_RES = FOREST_C^*∗*^FOREST_AREA^*∗*^f_re_r^*∗*^(20-temp)
  AQUACULTURE = AQU_AREA^*∗*^CE_AQU
  PC = car_r^*∗*^NI^*∗*^CE_car
  DESALINATION = (1^*∗*^SD1+2^*∗*^SD2+3^*∗*^SD3+4^*∗*^SD4+ 5^*∗*^SD5+365^*∗*^NI)^*∗*^PER_WC^*∗*^CE_DESALInation
  SHOPPING = (1^*∗*^SD1+2^*∗*^SD2+3^*∗*^SD3+4^*∗*^SD4+5^*∗*^ SD5)^*∗*^CE_SHOPPING
  WA = (1^*∗*^SD1+2^*∗*^SD2+3^*∗*^SD3+4^*∗*^SD4+5^*∗*^SD5)^*∗*^ CE_WA
  VISITING = (1^*∗*^SD1+2^*∗*^SD2+3^*∗*^SD3+4^*∗*^SD4+5^*∗*^ SD5)^*∗*^CE_VISITING
  FISHING = (1^*∗*^SD1+2^*∗*^SD2+3^*∗*^SD3+4^*∗*^SD4+5^*∗*^SD5)^*∗*^ CE_fishing
  HOTEL = (0^*∗*^SD1+1^*∗*^SD2+2^*∗*^SD3+3^*∗*^SD4+4^*∗*^SD5) ^*∗*^hotel__r^*∗*^CE_HOTEL
  PH = (0^*∗*^SD1+1^*∗*^SD2+2^*∗*^SD3+3^*∗*^SD4+4^*∗*^SD5)^*∗*^ CE_PH^*∗*^ph_r
  FERRY = NV^*∗*^dis_ferry^*∗*^CE_FERRY
  RM = (1^*∗*^SD1+2^*∗*^SD2+3^*∗*^SD3+4^*∗*^SD4+5^*∗*^ SD5)^*∗*^moto_r^*∗*^PD_moto^*∗*^CE_moto
  CAR = (1^*∗*^SD1+2^*∗*^SD2+3^*∗*^SD3+4^*∗*^SD4+5^*∗*^SD5)^*∗*^ PD_PC^*∗*^pc_r^*∗*^CE_PC
  SSB = (1^*∗*^SD1+2^*∗*^SD2+3^*∗*^SD3+4^*∗*^SD4+5^*∗*^SD5)^*∗*^ PD_SSD^*∗*^ssd_r^*∗*^CE_SSB
  OUTFLOWS:
  W_PHO = WETLAND_C^*∗*^w_growth_r^*∗*^TIDAL_ AREA^*∗*^1.05$\hat{\;\;}$(20-temp)^*∗*^(rade/(rade+6)^*∗*^690)
  F_PHO = FOREST_C^*∗*^f_growth_r^*∗*^FOREST_AREA^*∗*^ 1.05$\hat{\;\;}$(20-temp)^*∗*^(rade/(rade+6)^*∗*^690)
  ADSORPTION = SOIL_C^*∗*^ads_r^*∗*^(20-temp)
  SG = AQU_AREA^*∗*^GROWTH_R^*∗*^SHEEL_C_R/0.27
  FOREST_C(t) = FOREST_C(t - dt) + (F_PHO - F_RES - DECOMPOSION)^*∗*^dt
  INIT FOREST_C = 0
  INFLOWS:
  F_PHO = FOREST_C^*∗*^f_growth_r^*∗*^FOREST_AREA^*∗*^ 1.05$\hat{\;\;}$(20-temp)^*∗*^(rade/(rade+6)^*∗*^690)
  OUTFLOWS:
  F_RES = FOREST_C^*∗*^FOREST_AREA^*∗*^f_re_r^*∗*^(20-temp)
  DECOMPOSION = FOREST_C^*∗*^dec_r^*∗*^(20-temp)
  SHELL_C(t) = SHELL_C(t - dt) + (SG)^*∗*^dt
  INIT SHELL_C = 0
  INFLOWS:
  SG = AQU_AREA^*∗*^GROWTH_R^*∗*^SHEEL_C_R/0.27
  SOIL_C(t) = SOIL_C(t - dt) + (ADSORPTION + DECOMPOSION) ^*∗*^ dt
  INIT SOIL_C = 0
  INFLOWS:
  ADSORPTION = SOIL_C^*∗*^ads_r^*∗*^(20-temp)
  DECOMPOSION = FOREST_C^*∗*^dec_r^*∗*^(20-temp)
  TCH4(t) = TCH4(t - dt) + (WET_CH4_RELEASE + UNUSED_CH4)^*∗*^dt
  INIT TCH4 = 0
  INFLOWS:
  WET_CH4_RELEASE = 0.1^*∗*^WETLAND_C^*∗*^(20-temp)
  UNUSED_CH4 = WASTE_CH4^*∗*^CH4_USE_R
  WASTE_CH4(t) = WASTE_CH4(t - dt) + (WASTE - UNUSED_CH4)^*∗*^dt
  INIT WASTE_CH4 = 0
  INFLOWS:
  WASTE = (1^*∗*^SD1+2^*∗*^SD2+3^*∗*^SD3+4^*∗*^SD4+5^*∗*^ SD5+365^*∗*^NI)^*∗*^per_waste^*∗*^pCH4_WASTE
  OUTFLOWS:
  UNUSED_CH4 = WASTE_CH4^*∗*^CH4_USE_R
  WETLAND_C(t) = WETLAND_C(t - dt) + (W_PHO - W_RES - LITTER_TO_SEA - WET_CH4_RELEASE)^*∗*^dt
  INIT WETLAND_C = 0
  INFLOWS:
  W_PHO = WETLAND_C^*∗*^w_growth_r^*∗*^TIDAL_ AREA^*∗*^1.05$\hat{\;\;}$(20-temp)^*∗*^(rade/(rade+6)^*∗*^690)
  OUTFLOWS:
  W_RES = w_re_r^*∗*^TIDAL_AREA^*∗*^(20-temp)^*∗*^WETLAND_C
  LITTER_TO_SEA = 0.5^*∗*^WETLAND_C
  WET_CH4_RELEASE = 0.1^*∗*^WETLAND_C^*∗*^(20-temp)
  ads_r = 0.1
  AQU_AREA = 0.005^*∗*^INSHOR_AREA
  car_r = 0.1
  CE_AQU = 50000
  CE_car = 5400000
  CE_DESALInation = 2784
  CE_FERRY = 106
  CE_fishing = 1670
  CE_HOTEL = 7900
  CE_moto = 0
  CE_PC = 63
  CE_PH = 1619
  CE_SHOPPING = 344
  CE_SSB = 40
  CE_VISITING = 417
  CE_WA = 15300
  CH4_USE_R = 1
  dec_r = 0.15
  dis_ferry = 140
  FOREST_AREA = 0.53^*∗*^LAND_AREA
  f_growth_r = 0.1
  f_re_r = 0.2
  GROWTH_R = 1500000
  hotel__r = 0.3
  INSHOR_AREA = 1500
  LAND_AREA = 6.62
  moto_r = 0.2
  NI = 10000
  NV = 1000000
  pCH4_WASTE = 80000
  pc_r = 0.1
  PD_moto = 6
  PD_PC = 20
  PD_SSD = 14
  per_waste = 1.1
  PER_WC = 1.2
  ph_r = 0.7
  rade = 1.2
  SD1 = 0.1^*∗*^NV
  SD2 = 0.2^*∗*^NV
  SD3 = 0.5^*∗*^NV
  SD4 = 0.1^*∗*^NV
  SD5 = 0.1^*∗*^NV
  SHEEL_C_R = 0.95
  ssd_r = 0.7
  temp = 8
  TIDAL_AREA = 0.14^*∗*^LAND_AREA
  w_growth_r = 0.1
  w_re_r = 0.2

## 4. Result

Table 6 shows the general result of the carbon cycle process of Gouqi Island in 2014, Figure 4 indicates the island has achieved a positive removal of CO_2_ at the amount of 15540 t. Based on the average data from 2011 to 2013, the carbon credit of Gouqi Island is −14460 t, 36780 tons of carbon is released to atmosphere with the form of CO_2_, and 51240 tons of carbon is captured by the ecosystem. The three major resources of carbon emission are transportation and tourism development (the emission from tourist transportation are excluded) and seawater desalination, which contribute 35.2%, 20.0%, and 18.7% of the total carbon emission. The process of soil respiration, including soil microbial respiration, root respiration, soil animal respiration, is also a considerable carbon source, which contributes 17.1% of the total carbon emission.

Forest ecosystem and wetland ecosystem contribute to 7.3% and 11.4% of the total carbon capture. The island of Gouqi is named after a kind of major bush Chinese wolfberries* Lycium chinense* (in Chinese it is called Gouqi), which covers 53% percent of the mainland on the island. The ability of carbon sink of the bush forest is weaker than the evergreen broad-leaved forest at the same latitude [20]. Rock estuary is the major constitutes of the wetland ecosystem on the island, and thus its ability of carbon sink is weaker than the tidal marsh at the same latitude [21].

Mussel culturing industry is the most important carbon sink on the carbon cycle model of the island, which contributes to 50.3% of the carbon exchange and 81.2% among the total carbon sink. Shellfish utilize dissolved HCO_3_^−^ from seawater carbon to generate calcium carbonate (CaCO_3_) shells after Ca^2+^ + 2HCO_3_^−^ = CaCO_3_ + CO_2_ + H_2_O [22]. As the output of the primary industry and major input for the secondary industry on the island, mussel itself plays an important role for the carbon neutrality on the island.

## 5. Discussion

The application of the model as the environmental management tool could give the advices on the following questions.

**Figure 4:**
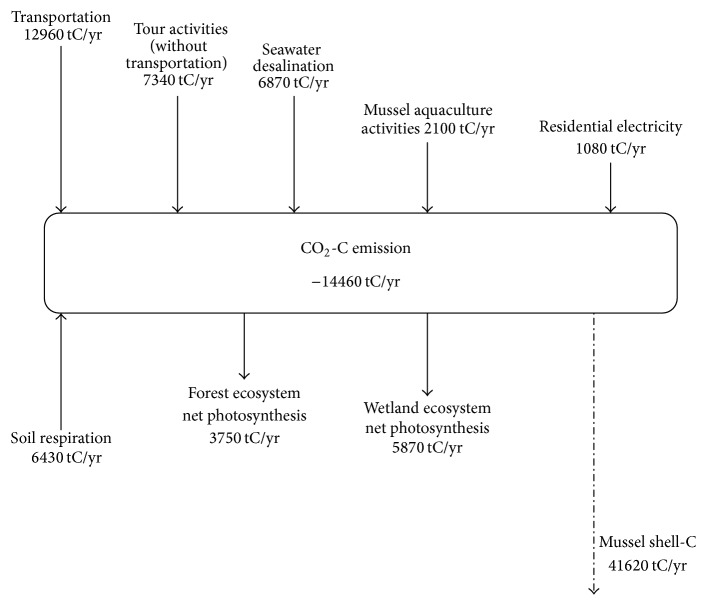
The carbon balance based on the results of the social-ecological process carbon cycle model on Gouqi Island.

*(1) What Will Happen with the Carbon Balance If the Island Transportation Becomes Fossil Fuel-Free?* The coastal line around the island is 7.1 km; it is possible that we use electricity as the power of transportation in place of fossil fuels. As illustrated in Figure 4, if we use electricity that generates no carbon emission, 1890 tCyr^−1^ can be saved. Further, if the ferry can be driven by electricity, 12960 tCyr^−1^ can be reduced, which can decrease 40.4% of the total carbon emission of the island. 

*(2) What Will Happen with the Carbon Cycle If the Island Develops More Carbon Sink Industry?* As the above analysis, the carbon credits of the island were negative because mussel plays a critical role of carbon capturing during its growth, which contributes more than 50% of the total carbon removal. As the mussel culturing has a high requirement of sea currents, the sea area around the island is limited and can hardly extend new area for mussel culturing; what is more, the frequent hurricane is a threat to the industry. Macroalga is less restricted by sea currents and has significant wave-breaking effect and carbon-capturing effect at the rate 3350 tC/yr·km^2^. If ecological project of marine carbon sink is introduced to the island, the growing condition of mussel can be safer, and the carbon credit of the island can be further improved. Assuming that we culture large alga beyond the mussel culturing area at a scale of three times of current mussel area, the total carbon credit will improve 70%. 

*(3) What Will Happen with the Carbon Cycle If the Island Conducts Sustainable Tourism Strategy?* Tourism, characterized by the rapidly increasing tourists number, is now a strategic direction for Gouqi Island with great potential on both economic and social perspective [23]. Meanwhile, tourism has been identified as an important contributor to carbon emission, accounting for a share of about 5% of global emissions CO_2_ [24], especially on the island-featured destination which relies heavily on fossil fuels to provide transportation, accommodation, food, and tourism activities [25]. In this article, we focus on the environmental influence of tourism considering the carbon neutrality on the island; thus we just consider the transportation between the island and the major ferry ports and on the island. If we want to discuss the carbon credit of the sustainable tourism on the island, it is necessary to consider the carbon emission inbound and outbound transportation [26]. It is necessary for the island to consider the components of carbon cycle on the island at different scenarios according to different tourism development strategy, for instance, tourists scale, tourist-guest source marketing, tourism attractions, total area and contents for tourist activity, tourism infrastructure construction, tourist accommodation upgrading, and so forth. This will help the island to achieve carbon neutrality balancing the economic and ecological goal.

## 6. Conclusion

Sustainable tourism destination has been an important research question, particularly for the island destination which has complex social-ecological interaction. We develop a carbon cycle model of Gouqi Island illustrating the carbon credit and its components. With the average data from 2011 to 2013, we can calculate that the carbon credit of the island in 2014 was −14460 tC/yr. The main sources of carbon emission are transportation, tourist activity, and seawater desalination, while the major carbon sink is mussel culturing which contributes to more than 50% of the carbon capture. We then discuss the carbon credit under different scenarios, for instance, the use of fossil-free energy in transportation and the development of ecological project such as large alga.

The carbon cycle model as a tool for environmental management model can extend to the development of industrial strategic design, by the way of modifying the coefficient or process in the model according to different strategy.

## Figures and Tables

**Table 1:** The basic information of Gouqi Island.

Gouqi Island	Value
Land area (km^2^)	6.62
Sea area (km^2^)	1,600
Population	10,470 (2009a)
GDP (million yuan RMB)	912 (2012a)
Primary industry (million yuan RMB)	301 (2012a)
Secondary industry (million yuan RMB)	198
Tertiary industry (million yuan RMB)	413
Forest coverage rate (%)	53
Tidal wetland area (km^2^)	0.92
The number of tourists	210,400

**Table 2:** State variables of the model, all expressed as tons of carbon on Gouqi Island as *f*(*time*).

Symbol	Description	Unit
CO_2_(t)	Carbon dioxide as *f*(*time*)	g CO_2_/yr
Forest C(t)	Carbon in forest ecosystem as *f*(*time*)	g C/yr
WETLAND_C(t)	Carbon in wetland ecosystem as *f*(*time*)	g C/yr
SHELL_C(t)	Carbon in aquaculture mussel shell as *f*(*time*)	g C/yr
SOIL_C(t)	Carbon in soil sink as *f*(*time*)	g C/yr
TCH_4_(t)	Total methane released as *f*(*time*)	g CH_4_/yr
WASTE_CH_4_(t)	Methane released from solid waste as *f*(*time*)	g CH_4_/yr

**Table 3:** Forcing functions of the model.

Symbol	Meaning
Temp	Temperature
Rade	Solar radiation
Nv	Number of visitors
Ni	Number of inhabitants
Land area	Land area of Gouqi Island
Inshore area	Sea area belonging to Gouqi Island management

**Table 4:** Processes of the model.

Process symbol	Meaning	Unit
W RES	CO_2_ released from respiration of plants in wetland ecosystem	g CO_2_
F RES	CO_2_ released from respiration of wetland plants in forest ecosystem	g CO_2_
AQUACULTURE	CO_2_ released from marine aquaculture industry	g CO_2_
PC	CO_2_ released from the use of private cars on the island	g CO_2_
DESALINATION	CO_2_ released from seawater desalination	g CO_2_
SHOPPING	CO_2_ released from tourist shopping	g CO_2_
WA	CO_2_ released from tourist water activity that used fossil fuels-driven motors	g CO_2_
VISITING	CO_2_ released from tourist sight-seeing on the island	g CO_2_
FISHING	CO_2_ released from tourist offshore angling	g CO_2_
HOTEL	CO_2_ released from tourist accommodation (hotel)	g CO_2_
PH	CO_2_ released from tourist accommodation (private home)	g CO_2_
FERRY	CO_2_ released from ferries that connect the island and outside	g CO_2_
RM	CO_2_ released from tourist transportation by rental motorcycles on the island	g CO_2_
CAR	CO_2_ released from tourist transportation by private car on the island	g CO_2_
SSB	CO_2_ released from tourist transportation by small shuttle bus on the island	g CO_2_
W PHO	CO_2_ captured by wetland plants through photosynthesis	g CO_2_
F PHO	CO_2_ captured by forest plants through photosynthesis	g CO_2_
ADSORPTION	CO_2_ captured by soil respiration	g CO_2_
SG	CO_2_ captured by mussel for shell growth during aquaculture	g C
DECOMPOSITION	Carbon entering the soil from forest vegetation litter	g C
WETCH_4_ RELEASE	CH_4_ released from wetland ecosystem	g CH_4_
UNUSED CH4	CH_4_ released from solid waste landfill that are not collected for further use	g CH_4_
WASTE	CH_4_ released from solid waste landfill	g CH_4_
LITTER TO SEA	Carbon entering marine ecosystem from wetland plant litter	g C

**Table 5:** Summary of the parameter symbols used in the model and the resources of the data. (SR = statistic report; FS = field survey; Q = questionnaire; R = reference.)

Abbreviation	Meaning	Unit	Source
Aqu area	Aquaculture area of mussel	km^2^	SR
Ads r	Adsorption rate of soil carbon pool	G Cm^−2^yr^−1^	R [12]
Car r	Possessing rate of private car	%	SR
CE AQU	CO_2_ released from aquaculture industry	g CO_2_km^−2^	FS
CE CAR	CO_2_ released from small private car per km	g CO_2_km^−1^	FS
CE DESALINATION	CO_2_ released through the desalination per ton of sea water	g CO_2_t^−1^	FS
CE FERRY	CO2 released from ferry per kilometer	g CO_2_km^−1^	FS
CE FISHING	CO_2_ released from sea fishing per hour	g CO_2_hr^−1^	FS
CE MOTO	CO_2_ released from motor bicycle per km	g CO_2_km^−1^	R [13]
CE HOTEL	CO_2_ released from hotel per day	g CO_2_/night	R [13]
CE Ph	CO_2_ released from private house per day	g CO_2_/night	R [13]
CE SHOPPING	CO_2_ released from tourist shopping per tourist per time	g CO_2_/visitor	R [13]
CE SSB	CO_2_ released from small shuttle bus	g CO_2_km^−2^	FS
CE VISITING	CO_2_ released from cultural tourism activities	g CO_2_hr^−1^	R [14]
CE WA	CO_2_ released from water tourism activities	g CO_2_hr^−1^	FS
CH4 UNUSE R	Unused rate of CH_4_ released from solid waste	%	SR
Dec r	Decomposition rate of forest litterfall	g Cm^−2^yr^−1^	R [14]
Dis ferry	Average driving distance of ferry	km	SR
Forest area	Coverage rate of forest	%	SR
F growth	Growth rate of forest	g Cm^−2^yr^−1^	R [14]
F re r	Respiration rate of forest	g Cm^−2^yr^−1^	R [14]
Growth r	Growth rate of mussel	g Cm^−2^yr^−1^	FS
NI	Number of inhabitants	/	SR
NV	Number of visitors	/	SR
Hotel r	Proportion of tourists choosing hotel	%	Q
Ph r	Proportion of tourists choosing private home	%	Q
Moto r	Proportion of tourists renting motorcycles on the island for transportation	%	Q
Pc r	Portions of tourists driving private cars on the island for transportation	%	Q
Ssd r	Portions of tourists taking small buses on the island for transportation	%	Q
PCH4 WASTE	CH_4_ released from solid waste of per kilogram	g CH_4_kg^−1^	R [13]
Pd moto	Average driving distance of motorcycles per day	km	Q
Pd pc	Average driving distance of private cars	km	Q
Pd ssd	Average driving distance of small buses	km	Q
Per waste	Average solid waste generated by per person	kg	SR
Per WC	Average amount of freshwater consumed by per person per day	t	SR
Rade	Average solar radiation	MJ m^−2^yr^−1^	R [14]
SD1	Proportion of tourists stay for one day	%	Q
SD2	Proportion of tourists stay for two days	%	Q
SD3	Proportion of tourists stay for three days	%	Q
SD4	Proportion of tourists stay for four days	%	Q
SD5	Proportion of tourists stay for more than five days	%	Q
SHELL C R	Proportion of carbon in per kilogram mussel	%	FS
Temp	Temperature	°C	SR
W growth r	Growth rate of wetland plants	g Cm^−2^yr^−1^	R [15]
W re r	Respiration rate of wetland plants	g Cm^−2^yr^−1^	R [15]

**Table 6:** General result of the carbon cycle model for Gouqi Island with the data of 2014.

Item	Carbon emission (t Cyr^−1^)	Carbon sink (t Cyr^−1^)
Ferry	11070	
Small shuttle bus	310	
Private car	1560	
Fishing	1300	
Visiting	320	
Water activity	1200	
Shopping	270	
Hotel	1220	
Private home	600	
Desalination	6870	
Mussel aquaculture activity	2100	
Shell aquaculture		41620
Soil respiration	6430	
Forest ecosystem		3750
Wetland ecosystem		5870
Solid waste	700	

## Appendix

### Questionnaire Sample

  Tourist No. —
  Duration of the trip: From — to —
  Day 1
    Accommodation type:
      □ Hotel
      □ Private home
      □ Others.
    Stay — hrs
    Transportation type:
      Site A to Site B (travel distance: — km),
      vehicle: —
      Site B to Site C (travel distance: — km),
      vehicle: —
      Site C to Site D (travel distance: — km),
      vehicle: —
    Activity:
      □ Sight seeing: — hrs;
      □ Fishing: —
  Day 2
  ⋮
  Day* N*
